# Cell therapy attempted as a novel approach for chronic traumatic brain injury – a pilot study

**DOI:** 10.1186/s40064-015-0794-0

**Published:** 2015-01-17

**Authors:** Alok Sharma, Hemangi Sane, Pooja Kulkarni, Jayanti Yadav, Nandini Gokulchandran, Hema Biju, Prerna Badhe

**Affiliations:** Department of Medical Services and Clinical research, NeuroGen Brain & Spine Institute, Stem Asia Hospital and Research Centre, Sector – 40, Plot No. 19, Palm Beach Road, Seawood (W), New Mumbai, 400706 India; Department Of Research & Development, NeuroGen Brain & Spine Institute, Stem Asia Hospital and Research Centre, Sector – 40, Plot No. 19, Palm Beach Road, Seawood (W), New Mumbai, 400706 India; Department Of NeuroRehabilitation, NeuroGen Brain & Spine Institute, Stem Asia Hospital and Research Centre, Sector – 40, Plot No. 19, Palm Beach Road, Seawood (W), New Mumbai, 400706 India

**Keywords:** Traumatic brain injury, Cellular therapy, Autologous, Bone marrow, Mononuclear cells, Intrathecal, PET CT scan

## Abstract

Traumatic brain injury is an injury to the brain parenchyma resulting from external factors such as vehicular accidents, falls, or sports injuries. Its outcome involves primary insult followed by a cascade of secondary insult, resulting in diffuse axonal injury further causing white matter damage. Surgical intervention targets the primary damage, whereas only few treatment alternatives are available to treat the secondary damage. Cellular therapy could be one of the prospective therapeutic options, as it has the potential to arrest the degeneration and promote regeneration of new cells in the brain. We conducted a pilot study on 14 cases who were administered with autologous bone marrow mononuclear cells, intrathecally. The follow up was done at 1 week, 3 months and 6 months after the intervention. The Functional Independence Measure scale, the SF-8 Health Survey Scoring and the disability rating scale were used as outcome measures. These scales showed a positive shift in scores at the end of 6 months. Improvements were observed in various symptoms, along with activities of daily living. Improvement in PET CT scan performed before and 6 months after the intervention in 3 patients corresponded to the clinical and functional improvements observed in these patients. The results of this study suggest that cell therapy may promote functional recovery leading to an improved quality of life in chronic TBI. Although the results are positive, the improvements after cell therapy are not optimal. Hence, additional multicenter, controlled studies are required to establish cell therapy as a standard therapeutic approach.

## Introduction

Traumatic brain injury (TBI) is defined as *“An insult to the brain not of the degenerative or congenital nature, but caused by an external physical force that may produce a diminished or altered state of consciousness which results in impairment of cognitive abilities or physical functions”* (Bunch and Jennifer 2000). It is one of the leading causes of morbidity and mortality in the world. It may result in temporary or permanent behavioral and/or emotional disturbances causing functional disability. The underlying mechanism of injury involves primary damage occurring at the time of impact and secondary damage which is initiated at this time but the clinical presentation is delayed. TBI leads to alterations in cerebral blood flow and oxygenation, cell death and generalized atrophy (Prins et al. 2013).

Very few therapeutic options are currently available to treat TBI. As the brain has a limited potential to regenerate the neurons, the intervention should aim at halting the degeneration and carrying out the process of regeneration (Horner and Gage 2000). Hence, cell therapy may be a prospective option. In experimental models, it has been observed that cell therapy improves the neurological functional outcome in TBI (Kim et al. 2010). Transplanted cells may replace the lost cells by proliferation and differentiation. They can also stimulate the endogenous repair by release of trophic factors (Hsu et al. 2013). To study the benefits of cell therapy in chronic TBI, we administered 14 patients with autologous bone marrow derived mononuclear cells (BMMNCs), intrathecally. BMMNCs contain a mixture of different types of progenitor cells. It possesses hematopoietic stem cells and stromal cells. These cells were chosen as they are easily obtainable, do not involve any ethical issues, and are immunologically safe (Morizane et al. 2013; Parr et al. 2007). These cells, on injection migrate to the damaged area and bring about the repair process. They differentiate into neurons, astrocytes, microglial cells and oligodendrocytes (Peter and Chen 2002).

### Rationale for using cell therapy for traumatic brain injury

Over the last few years, cell therapy has acquired significant interest as a potential treatment strategy for various neurological disorders such as stroke, cerebral palsy, autism, spinal cord injury, and traumatic brain injury (Sharma et al. 2012a; Sharma et al. 2012b; Sharma et al. 2012c; Sharma et al. 2012d; Sharma et al. 2013a; Sharma et al. 2013b; Sharma et al. 2013c; Zhang et al. 2013). A variety of cell types have been explored as potential source of transplantation for neurological disorders, including embryonic cells, bone marrow stem cells, neural stem cells, umbilical cord blood cells and induced pluripotent cells (Ul Hassan et al. 2009; Liu et al. 2000; Muotri 2010; Zhao et al. 2002). The underlying mechanism of cell therapy, to promote angiogenesis, axonal remodeling, neurogenesis and synaptogenesis, may help reverse the pathology of TBI.

## Materials and method

### Study design and patient selection

An open label clinical study was performed to demonstrate the effect of intrathecal autologous bone marrow mononuclear cells in chronic traumatic brain injury. The protocol of the study was reviewed and approved by The Institutional Committee for Stem Cell Research and Therapy (IC-SCRT) in accordance to the Indian Council of Medical Research (ICMR) guidelines. Fourteen TBI patients were selected based on the World Medical Association Helsinki Declaration for Ethical Principles for medical research involving human subjects (World Medical Association 2013). A written informed consent was obtained from the patients and their families depending on the patient’s cognitive status. The inclusion criteria were diagnosed cases of chronic TBI and age above 1 year. The exclusion criteria were presence of acute infections such as HIV/HBV/HCV, malignancies, bleeding tendencies, renal failure, severe liver dysfunction, severe anemia [Hb < 8], pregnancy, lactation, any bone marrow disorder and other acute medical conditions such as respiratory infection and pyrexia.

### Study protocol

The intervention included cellular therapy and neurorehabilitation. Cellular therapy included intrathecal administration of autologous bone marrow mononuclear cells and neurorehabilitation included physiotherapy, occupational therapy, speech therapy and psychological intervention. Patient was admitted for 6 days which included: Day 1 of pre-intervention assessment, Day 2 of bone marrow aspiration and administration of separated BMMNCs, Day 3 to Day 6 of multidisciplinary neurorehabilitation. Patients were followed up at 3 months, 6 months and yearly thereafter.

### Pre-intervention assessment

Before the intervention, every patient underwent a detailed neuro-evaluation by medical experts. Serological, biochemical and hematological tests were also performed. Functional independence of all the patients was evaluated using Functional Independence Measure (FIM). Electroencephalography (EEG), Magnetic Resonance Imaging (MRI) of brain, with Diffusion tensor imaging (DTI) and Positron Emission Tomography- Computed Tomography (PET-CT) brain scans were performed in all patients before the treatment.

### Bone marrow aspiration and isolation of bone marrow mononuclear cells

Granulocyte Colony Stimulating Factor (G-CSF) injections were administered 48 hours and 24 hours prior to the procedure as it stimulates and mobilizes the bone marrow stem cells (Yoon et al. 2007).

Approximately 80-100 ml bone marrow was aspirated from the anterior superior iliac crest under local anesthesia with or without mild sedation (depending on the case scenario), using the bone marrow aspiration needle. The bone marrow was collected in heparinized tubes. Bone marrow smears were stained with Leishman stain. Quantitative and qualitative analysis of bone marrow was performed. MNCs were separated by differential centrifugation. Bone marrow dilution is done with normal saline (ratio 1:1). Centrifugation is done at 440 × g rpm for 35 minutes in a swinging bucket rotar without brake at 20°C. MNCs are separated as a buffy coat. They are resusupended in 1 ml of normal saline after washing thrice with normal saline.

They were checked manually for viability using trypan blue dye and confirmed with propidium iodide dye in TALI (Life Technologies. Invitrogen). Average viability was found to be 97%. CD34+ counting was also performed by fluorescence activated cell sorting (FACS) using CD34 PE antibody.

### Administration of bone marrow mononuclear cells

The separated autologous BMMNCs (body weight × 10^6^) were immediately injected intrathecally using an 18 G Touhy needle and spinal needle between fourth and fifth lumbar vertebrae under local anesthesia with or without mild sedation (depending on the case scenario). Simultaneously, 20 mg/kg body weight methyl prednisolone in 500 ml Ringer Lactate was given intravenously to enhance survival of the injected cells.

### Neurorehabilitation

Every patient underwent a personalized neurorehabilitation program immediately after cellular therapy and was advised to continue as a home program. This included physiotherapy, occupational therapy, psychological therapy and speech therapy.

### Follow –up and outcome measure

Patients were followed up at 1 week, 3 months and 6 months after the intervention. A complete neurological evaluation was performed. All the outcome measures were scored before and 6 months after the intervention. FIM was repeated to monitor improvements in daily functional activities. SF-8 Health Survey Scoring was performed to assess overall health of the patients for their quality of life. Only 7 out of 14 patients were eligible for SF-8 test, due to their cognitive status. Disability rating scale (DRS) was performed on all the patients. Three patients chose to repeat 18-FDG PET-CT scan at the end of 6 months to monitor the functional metabolic changes in brain after the intervention.

## Results

The study included fourteen patients with chronic TBI. (Table 1) The median age of the study sample was 26 years (Range: 12 years to 65 years). The patients were followed up at 1 week, 3 months and 6 months while, 1 patient was lost to follow up. A detailed neurological evaluation was performed for every patient.

**Table 1:** **Demographical data**

**Demographic characteristics**	**Demographic group**	**Number of patients**
**Sex**	Male	11
	Female	3
**Age**	<20 years	5
	20-30 years	4
	>30 years	5
**Cause of trauma**	RTA	7
	Fall from Height	3
	Train accident	3
	Fall of heavy object	1
**Duration since injury**	<5 year	8
	>5 years	6
**History of seizures**	Present	7
	Absent	7

(Total Sample Size =14).

One week after the intervention, 5 out of 13 patients improved in speech, 3 improved in trunk and upper limb activity, oromotor activities, muscle tone, voluntary control, ambulation and gait pattern and posture, 2 improved in lower limb activity, balance and psychological status, and 1 improved in cognition, memory, ADLs and communication.

Three months after the intervention, 8 showed improvements in voluntary control, 7 in trunk and upper limb activity, 6 in speech, lower limb activity and ambulation and gait patterns, 5 in posture, 4 in muscle tone, balance, ADLs, psychological status, coordination and communication, 3 in oromotor activities and 2 in cognition and memory.

**Figure 1:**
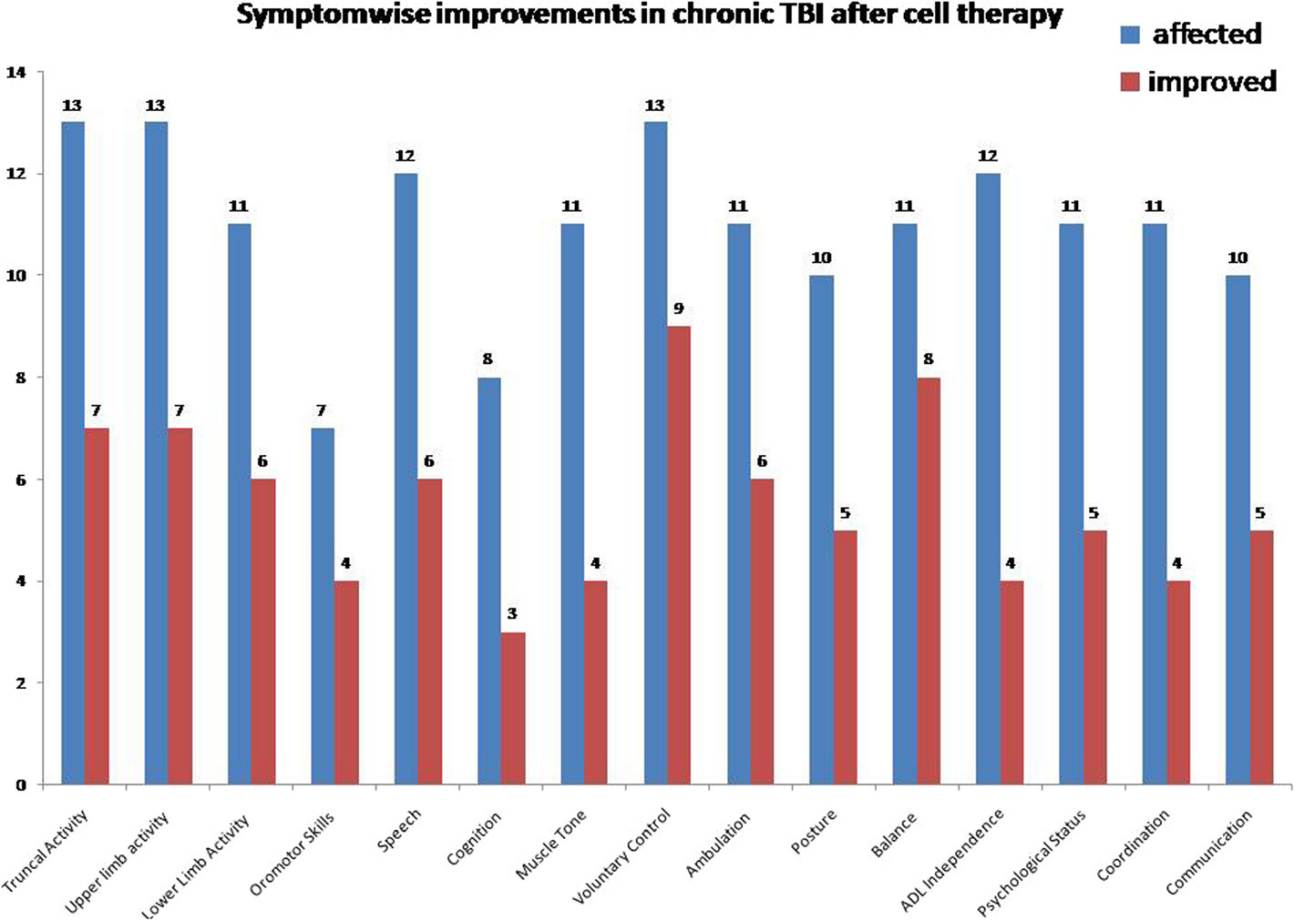
**Graph representing symptomatic improvements in traumatic brain injury after cell therapy.**

At the end of six months, a percentage analysis was carried out for improvement in every symptom (Figure 1). Amongst 13 patients, 73% showed improvement in balance, 69% in voluntary control, 60% in memory, 57% in oromotor activities, 55% in lower limb activity and ambulation and gait patterns, 54% in trunk and upper limb activity, 50% in speech, posture and communication, 45% in psychological status, 38% in cognition, 36% in muscle tone and coordination and 33% in ADLs.

**Figure 2:**
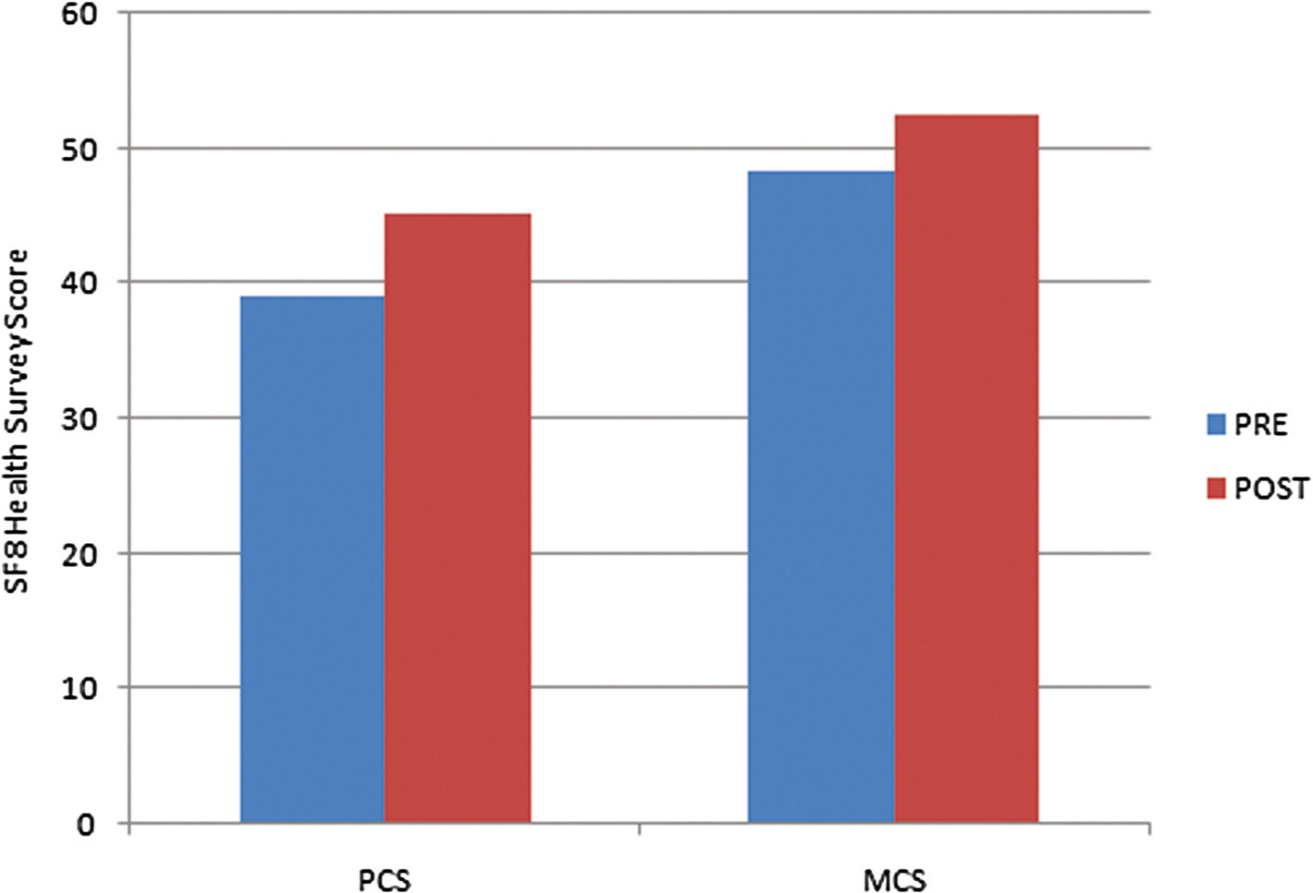
**Graph representing the mean values of pre and post physical components and mental components of SF8 health survey scoring.**

SF8 scale was performed on 7 patients who met the eligibility criteria of the test. Six months after the intervention, the mean physical component summary (PCS8) score improved from 39.11 to 45.27 and mean mental component summary (MCS8) from 48.44 to 52.55 (Table 2 and Figure 2). On the DRS, 6 out of 13 patients showed reduced scores while the scores remained the same in 7 cases. It was observed that these patients showed improved scores primarily in the cognitive component of the DRS.

**Table 2:** **SF8 scores before and after intervention**

**Patient no.**	**SF8 pre**		**SF8 post**	
	**PCS8**	**MCS8**	**PCS8**	**MCS8**
1	28.4	32.5	39.2	40.8
2	44.4	52.1	51.4	59.8
3	47.1	35.4	48.6	43.2
4	33.4	51.5	43	55.9
5	39	64.1	43.1	62.7
6	43.7	58.8	50.1	58.3
7	37.8	44.7	41.5	47.2

PET CT scans were repeated in 3 patients at the end of six months and they showed improved metabolism after intervention. The changes were consistent with the clinical and functional improvements demonstrated by these patients (Table 3).

**Table 3:** **Areas of the brain showing improved metabolism in PET CT scan and their correlation to the clinical function improvement**

**Patient**	**Areas of the brain showing improved metabolism in PET CT scan**	**Functions improved**
Patient 1	Parieto-Occipital Areas	Cognition, speech, sensation, orientation and visual perception
Patient 2	Cingulate Gyri	Emotion, attention, cognition, memory
	Amygdala	Emotional responses, memory, attention
	Frontal	Planning, Long term memory, emotions, speech, problem solving
	Temporal Lobes	Speech, memory
Patient 3	Amygdala	Emotional responses, memory, attention
	Cerebellum	Coordination, balance
	Cingulate Gyri	Emotion, attention, cognition, memory
	Basal Ganglia	Voluntary motor control, learning,cognition.
	Occipital Lobes	Vision and perception
	Parietal Lobe	Movement, orientation
	Temporal Lobes	Speech, memory
	Frontal Lobes	Planning, Long term memory, emotions, speech, problem solving
	Thalamus	Motor control, sensory functions

Ten out of 14 patients had an abnormal EEG before the intervention. Of these, 7 had a previous history of seizures and were on antiepileptic medication but, only one had an episode of seizure 3 months following intervention but this still warrants consideration as one of the adverse events of cell therapy.

## Discussion

The pathophysiology of TBI involves primary injury which is the immediate cause of physical or biomechanical effect of trauma. This is followed by a cascade of secondary events leading to excitotoxicity, increase in free radicals, cerebral ischemia, intracranial hypertension, hypo/hyperperfusion, impairment of cerebrovascular autoregulation and cerebral metabolic dysfunction (Smith et al. 1997). The cascade of events releases a number of neurochemical factors which are responsible for cellular death. This may result in regional or generalized atrophy, depending on the type and severity of injury (Bramlett and Dietrich 2007). Evidence suggests that diffuse injury is more common than focal injury and damage commonly occurs in the frontal and temporal lobes, corpus callosum, basal ganglia, hypothalamus, superior cerebellar peduncles, fornices and the hippocampus (Jorge and Starkstein 2005; Schwarzbold et al. 2008). Damage to these areas is primarily responsible for depression, mood disorders and other neurobehavioral and psychiatric deficits in TBI. In TBI, the white matter tracts are damaged due to the injury. This disrupts the cortical-subcortical pathways, further affecting the cognition of the patient. The greater the reduced white matter integrity, the greater is the cognitive deficit (Kraus et al. 2007). It is a challenge to repair the injured brain, due to the limited capacity of the brain to regenerate functional neurons. Currently, no clinical treatment is available to repair the diffuse axonal injury and reverse the pathologic cascade of cell death and improve neurobehavioral outcome. However, cell therapy has opened up new avenues for treatment strategies in various incurable neurological disorders including TBI. Different types of cells have been explored and amongst them bone marrow mononuclear cells are relatively safe and easily obtainable.

### Composition of bone marrow mononuclear cells

To demonstrate the effect of cell therapy on chronic TBI, we administered autologous bone marrow mononuclear cells in 14 patients. These cells are a heterogeneous mixture of hematopoietic cells, mesenchymal cells, very small embryonic like cells (VSELs) and endothelial progenitor cells. It has been observed that use of BMMNCs is more successful than the sub fractionated cell preparations (Lawall et al. 2010).

### Mechanism of action of bone marrow mononuclear cells

Cell therapy mimicks the natural repair process in TBI. In acute TBI the endogenous stem cells are activated as a natural process (Ahmed et al. 2014). These cells migrate towards the site of injury and carry out the repair process. However, the number of these activated cells are less in the chronic phase. Therefore, in chronic phase, cell therapy is a mode of increased supply of stem cells for repair.

Evidence suggests that these cells protect and repair the damaged central nervous system via multiple mechanisms. The transplanted cells survive and migrate towards the damaged tissues and promote functional recovery. They proliferate and differentiate into various cells including neural cells, oligodendrocytes, etc (Sanchez-Ramos et al. 2000). In TBI, the loss of myelin disrupts the signal transduction and damages the axons. The oligodendrocytes help in remyelination of the damaged axons in the injured brain and repair the neural connections. Bone marrow cells also produce various growth factors and neurotrophic factors such as brain-derived neurotrophic factor (BDNF), nerve growth factor (NGF), vascular endothelial growth factor (VEGF), basic fibroblast growth factor (bFGF), and hepatocyte growth factor (HGF), which stimulate the endogenous neuroprotection and repair (Zhong et al. 2003).

### Route of administration

To augment the effect of these cells, it is important to administer them through an optimal route of transplantation. In this study, we transplanted the cells intrathecally as they are minimally invasive and are more targeted than intravenous transplantation. Although direct intracerebral injection may be the most efficient route, it involves an invasive procedure which could result in secondary damage. Intravenous transplantation is also minimally invasive, but studies have shown that when these cells are delivered through this route they are initially trapped in the lungs and the number of cells reaching the target tissue is questionable (Fischer et al. 2009).

### Clinical outcome of this study

Our study population was followed up at 1 week, 3 months and 6 months after the intervention. We observed significant symptomatic improvements, starting from one week to 6 months after intervention. According to Park et al, the immediate functional improvements observed one week after the intervention may be due to the neuroprotective effects of neurotrophic factors produced by the transplanted cells rather than integration of stem cells and replacement of lost tissues (Park et al. 2006). Along with major symptoms of chronic TBI shown in Figure 1, improvement was observed in fine motor skills of 5 patients, attention and concentration of 3 patients, socialization skills of 2 patients, sensation and contractures/deformities of 1 patient. In addition, 1 patient with right side facial muscle paralysis also showed improvement after intervention.

Three patients showed an increase in FIM score, indicating improvement in daily functional activities. SF8, a health related quality of life scale, was performed on the eligible 7 patients. It is a brief questionnaire of 8 questions assessing physical and mental health of the affected patient. On SF8 scale, all patients showed improvement in physical components while 5 in mental component indicating improved quality of life. On DRS, 6 out of 13 patients showed reduced scores indicating improved functions. It was observed that the patients showing reduced scores had severe TBI while, the patients who showed no change had mild TBI. The lack of measured improvement in the mild TBI patients may be due to a lack of sufficient sensitivity of the DRS instrument, given that the pre-intervention scores in the mild TBI patients were near normal. Nonetheless, these outcome measures suggest that cell therapy may promote functional recovery leading to an improved quality of life in chronic TBI.

### PET CT scan findings

The PET CT scans provide measures of brain glucose metabolism using tracer [18F] fluorodeoxyglucose (FDG). The brain glucose metabolism indirectly correlates with the function of the neurons. Hypometabolism indicates hypofunctionality and hence, improvement in function will be seen as increased metabolism (FDG uptake). A limited number of studies have been performed with FDG-PET in TBI. Previous studies have shown decreased metabolism in the frontal cortex, temporal cortex, thalamus, and cerebellum areas after TBI (Kato et al. 2007; Nakashima et al. 2007). In this study, the patients who underwent PET CT scan 6 months post intervention showed significant changes as shown in supplemental table (Table 3). These changes corresponded to the symptomatic improvement. The PET CT scan records the cellular changes in the form of metabolism. Hence, for future studies, PET CT scan should be considered, not only for diagnosis, but also to monitor effect of cell therapy in TBI.

### Adverse events

This study also evaluated the adverse events associated with cell therapy. Out of 7 patients with an abnormal EEG and a history of seizures pre-intervention, only 1 patient had an episode of seizure within 3 months after intervention. The episode of seizures could be due to activation of epileptogenic foci or abnormal innervation during neurogenesis. Seizures, as adverse events, have been recorded earlier in studies of stroke and autism (Sharma et al. 2013c; Lee et al. 2010), Hence its possibility in TBI cannot be eliminated. Future detailed study will include an in-depth analysis.

### Limitations and future directions

One of the major limitations of this study is that it is an uncontrolled study with a small study population. Due to lack of control group, we have to be judicious about directly attributing improved scores solely to cell therapy as rehabilitation may have also contributed to the results in this study. There are limited number of clinical studies demonstrating the benefits of cell therapy in traumatic brain injury, so, this study supplements the available information and may help to design larger, multicentre, controlled studies.

Future studies should identify the ideal cells for therapeutic use, along with the ideal route of administration. The optimum quantity of cells, frequency of doses and the time interval between consecutive doses should be established. It is important to determine the timing of cell transplantation as in acute phase of injury; inflammation and pathological metabolic changes make the endogenous environment inhospitable for the injected cells making their survival difficult (Morganti-Kossmann et al. 2002). In addition, chronic effects may include gliotic changes, which may affect the efficacy of cell therapy. Another important area of concern is to monitor the changes in the brain and the repair process. In this study, PET CT scan was repeated in only 3 patients to monitor the metabolic changes in the brain after intervention. An objective method should be studied for this purpose. Various experiments need to be conducted and compared to discover the precise combination that will exhibit optimal results in chronic TBI.

## Conclusion

Our results suggest that cell therapy in combination with neurorehabilitation has a potential to reverse the damage occurred in the brain after chronic TBI. It addresses the diffused nature of injury, which a surgical intervention may not tackle. The resulting angiogenesis, along with endogenous repair, may improve the blood flow and oxygen supply to the injured areas of the brain, further leading to improved functional outcome and enhanced quality of life. As this is a pilot study, demonstrating the effect of cellular therapy in chronic TBI, more evidence will be required in order to establish cell therapy as a therapeutic modality.
